# Supplementary material for: Ethnobotanical Survey of Plants Used by Subsistence Farmers in Mitigating Cabbage and Spinach Diseases in OR Tambo Municipality, South Africa
Source: Plants (Basel). 2022 Nov 24;11(23):3215. doi: 10.3390/plants11233215 (PMC9741191; doi:10.3390/plants11233215)
Supplement: Supplementary file 1 [file plants-11-03215-s001.zip › plants-2025087-supplementary.pdf]

Table S1. Pictures of common diseases affecting cabbage and spinach in Eastern Cape, South Africa.

**CABBAGE DISEASES**	
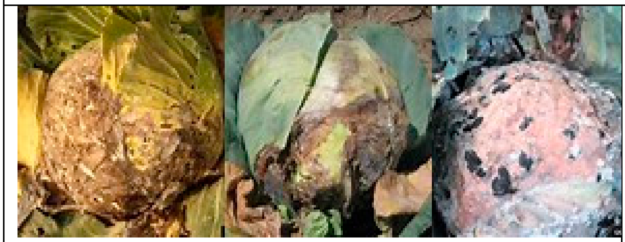<br>Sclerotinia = C1	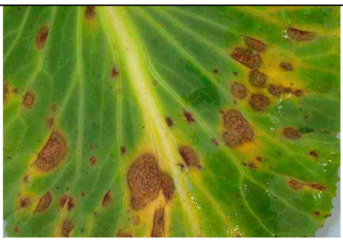<br>Ring spot = C2
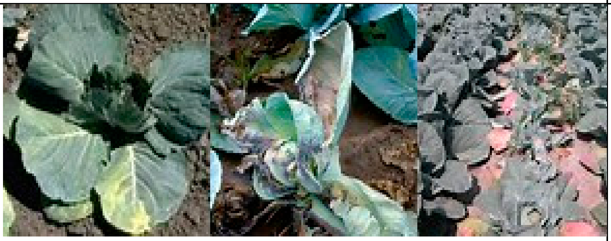<br>Fusarium wilt = C3	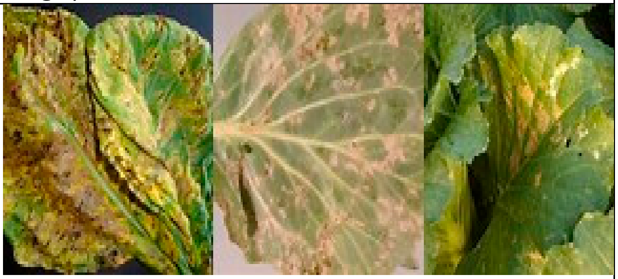<br>Downy Mildew = C4
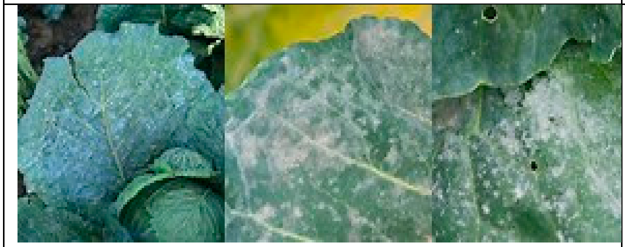<br>Powdery Mildew = C5	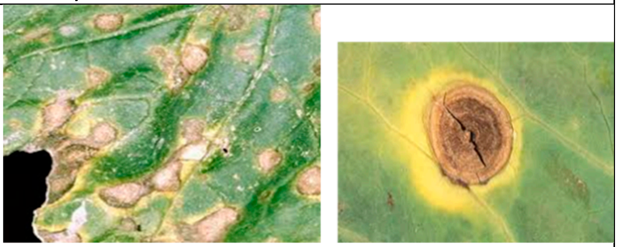<br>Alternaria leaf spot = C6
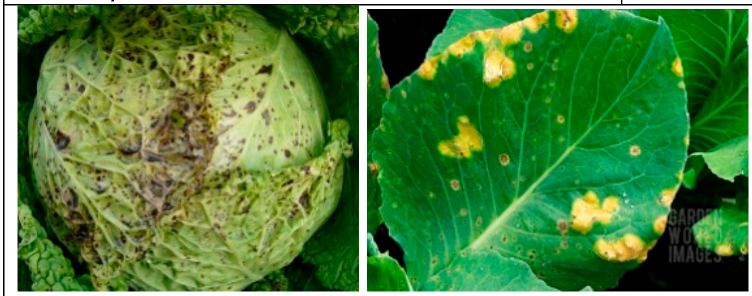<br>Bacterial spot = C7	

C8= Root rot

C9 = Black rot

SPINACH DISEASES

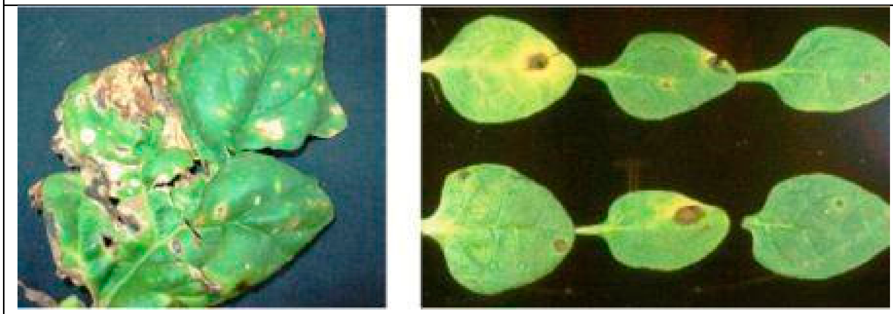

Anthracnose = S1

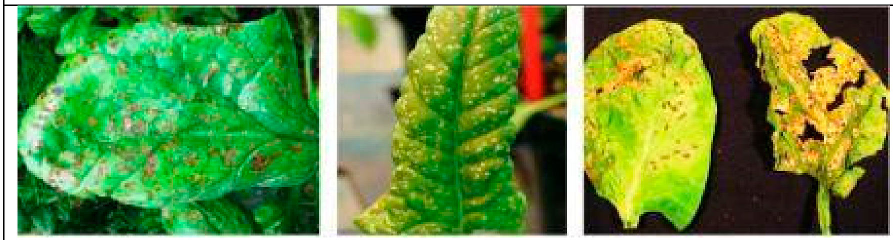

Cladosporium leaf spot = S2

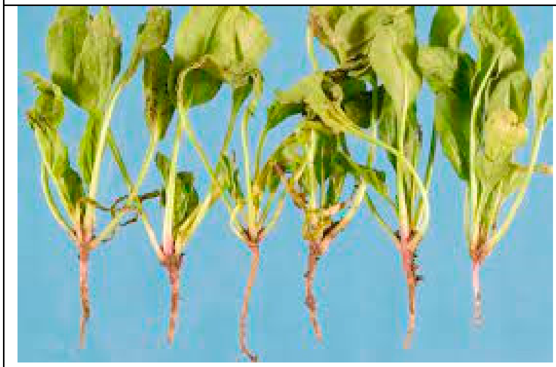

Damping off and root rot = S3

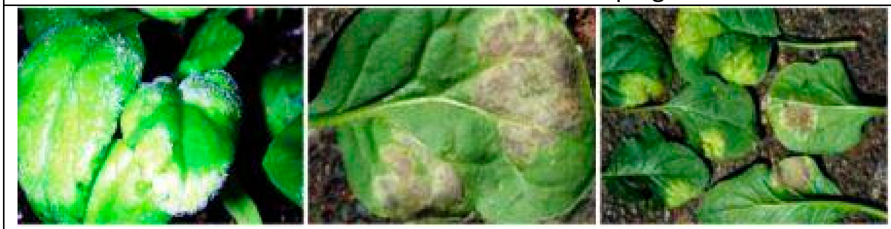

Downy mildew = S4

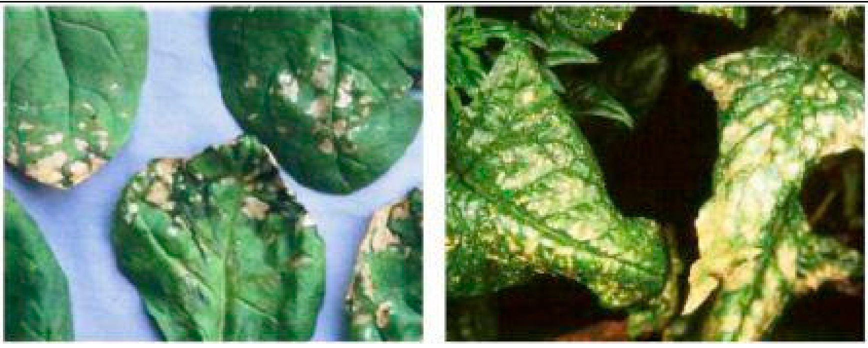

Stemphylium leaf spot = S5
